# An uncommon presentation of COVID-19: concomitant acute pulmonary embolism, spontaneous tension pneumothorax, pneumomediastinum and subcutaneous emphysema (a case report)

**DOI:** 10.11604/pamj.2021.39.26.29178

**Published:** 2021-05-10

**Authors:** Zakariae Belarbi, Falmata Laouan Brem, Siham Nasri, Skiker Imane, El Ouafi Noha

**Affiliations:** 1 Department of Cardiology, Mohammed VI University Hospital, Faculty of Medicine and Pharmacy of Oujda, Mohammed First University, Oujda, Morocco,; 2 Department of Radiology, Mohammed VI University Hospital, Mohammed First University, Oujda, Morocco,; 3 Epidemiological Laboratory of Clinical Research and Public Health, Oujda, Morocco

**Keywords:** COVID-19, spontaneous tension pneumothorax, pneumomediastinum, subcutaneous emphysema, case report

## Abstract

The presenting symptoms and features of COVID-19 are non-specific and may be extrapulmonary complications such as thrombotic disorders but also pneumothorax, pneumomediastinum and subcutaneous emphysema; which are well-known complications of mechanical ventilation. Nevertheless, pneumothorax and/or pneumomediastinum, could complicate the course of a COVID-19 disease even in the absence of barotrauma involved. Herein, we report the case of a 55-year-old man with a previous history of erythroblastopenia due to thymoma admitted for COVID-19-related acute respiratory distress syndrome (ARDS) who simultaneously developed spontaneous tension pneumothorax, pneumomediastinum, subcutaneous emphysema and acute bilateral pulmonary embolism as presenting features of COVID-19 while on high-flow nasal cannula. This rare case highlights the importance of screening for other coexisting alternative diagnoses at the initial presentation of a patient suspected of COVID-19.

## Introduction

Since December 2019, the novel coronavirus disease (COVID-19) has become very challenging for medical care. The presenting clinical features of COVID-19 are mainly respiratory but non-specific [1] and maybe complications such as thrombotic disorders [2] and, more rarely, other extrapulmonary complications such as pneumothorax, pneumomediastinum [3,4]. Mechanical ventilation due to intubation is a well-known cause of both pneumothorax and pneumomediastinum [5]. Nevertheless, pneumothorax and/or pneumomediastinum, could complicate the course of a COVID-19 disease even in the absence of barotrauma involved [6]. Herein, we report the case of a 55-year-old man with a previous history of erythroblastopenia due to thymoma admitted for COVID-19-related acute respiratory distress syndrome (ARDS) who developed simultaneously spontaneous tension pneumothorax, pneumomediastinum, subcutaneous emphysema, and acute bilateral pulmonary embolism as presenting features of COVID-19 while on high-flow nasal cannula (HFNC).

## Patient and observation

A 55-year-old man with a previous history of erythroblastopenia due to thymoma having undergone thymectomy followed by radio-chemotherapy and long-term corticosteroid therapy presented to the emergency department (ED) of our center after 15 days of fever, dry cough, asthenia, myalgia, headache with marked dyspnea. The patient was hemodynamically stable (blood pressure at 140/70mmhg, a heart rate at 80 beats/min) with an increased breathing (tachypnea 25 breaths/minute, oxygen saturation at 70% in room air), and a high temperature (38.2°C). The physical examination revealed subcutaneous emphysema in the chest and neck. Laboratory results showed leukocytosis (white blood cells at 28830 cells/μL), lymphopenia (280 cells/μL); elevated levels of inflammatory markers (C-reactive protein (CRP) of 150 mg/l, a high level of ferritin at 35674.76 ng/mL, a high fibrinogen level at 6.2g/L), a normal procalcitonin level of 0.46 ng/mL; a high D-Dimer level (21960ng/mL), and normal renal function. Arterial blood gas performed on high-flow nasal cannula (HFNC) 15L/min, showed a respiratory alkalosis (pH at 7.52, partial pressure of carbon dioxide (PCO_2_) at 31.6mmhg; HCO_3_ at 26.1mmhg) with hypoxemia (partial pressure of oxygen (PO_2_) at 49mmhg; SpO_2_ (oxygen saturation) at 89%).

**Figure 1:**
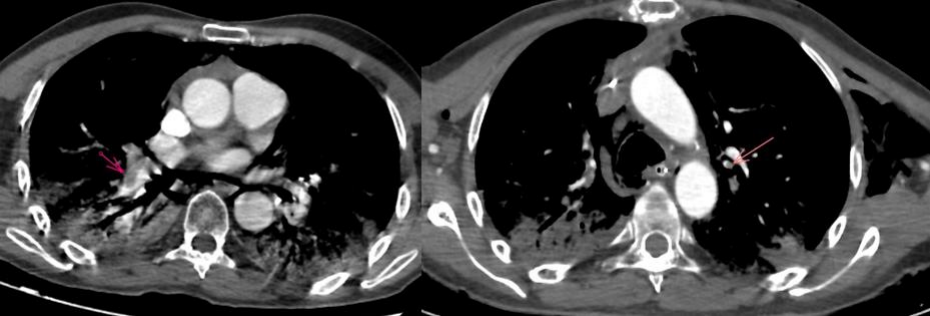
computed tomography pulmonary angiography in axial lung window showing acute bilateral pulmonary embolism (red arrows)

**Figure 2:**
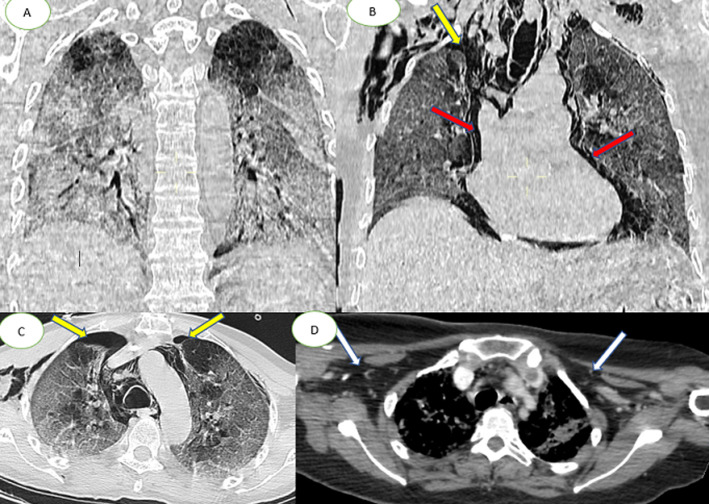
chest CT scan in coronal (A, B) axial (C, D) and lung parenchymal windows showing subpleural ground-glass opacities associated with multiple areas of consolidation as well as the presence of bilateral pneumothorax, (yellow arrows) pneumomediastinum, (red arrows); extensive bilateral subcutaneous emphysema (white arrows)

**Figure 3:**
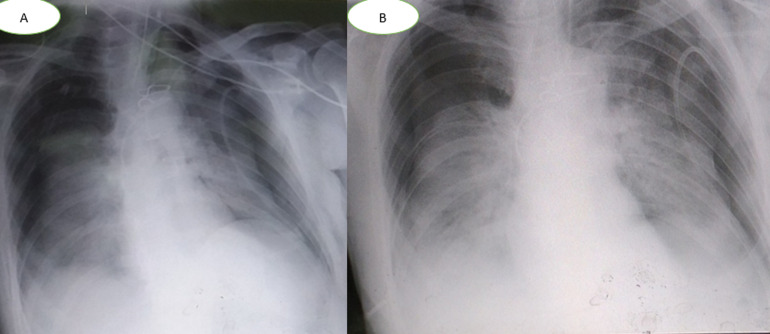
a chest X-ray demonstrating a return to the lung wall on the left side and the persistence of pneumothorax on the right side (A) and control after re-drainage (B)

The patient tested positive for SARS-CoV-2 reverses transcriptase-polymerase chain reaction test (RT-PCR) performed on nasopharyngeal swabs. According to these clinical and biological findings, a computed tomography pulmonary angiography (CTPA) was performed, which showed acute bilateral pulmonary embolism (Figure 1). Chest computed tomography (CT) scan also revealed subpleural ground-glass opacities associated with multiple areas of consolidation suggestive of COVID-19 pneumonia as well as the presence of bilateral pneumothorax, pneumomediastinum, and extensive bilateral subcutaneous emphysema (Figure 2A, B, C, D). The patient was transferred to the intensive unit care and received treatment with azithromycin 250mg once a day, ceftriaxone 2g once a day, ciprofloxacin 400mg twice a day, hydrocortisone, tocilizumab unique dose, therapeutic anticoagulation with low molecular weight heparin (LMWH), and supplemental oxygen on high-flow nasal cannula (HFNC). The pneumothorax required bilateral drainage with bubbling, and the pneumomediastinum was managed conservatively. However, the patient further deteriorated with acute respiratory failure, which required intubation with mechanical ventilation in volume-controlled mode controlled (the fraction of inspired oxygen (FIO_2_) at 100%, and positive end-expiratory pressure (PEEP) of 8cm H_2_0 was applied. A chest X-ray (Figure 3A) showed the pneumothorax persistence on the right side. He benefited from a re-drainage by a drain of Joly 24 with X-ray control (Figure 3B). Unfortunately, he deceased from respiratory failure 10 days after admission.

## Discussion

To the best of our knowledge, this is the first reported case of a patient with COVID-19 pneumonia with concomitant spontaneous pneumothorax (SPP), pneumomediastinum (SPM), subcutaneous emphysema (SCE), and acute bilateral pulmonary embolism as the initial presentation. Indeed, there have been only a few reported cases of patients who spontaneously developed pneumothorax, pneumomediastinum, subcutaneous emphysema in the context of COVID-19 [7,8] and no presentation of this association with acute pulmonary embolism have been previously described. However, there has been a single report of a COVID-19 patient who developed spontaneous tension pneumothorax and acute pulmonary emboli [9]. Therefore, SPM and SPP incidence in patients with COVID-19 pneumonia remains undefined. Depending on whether or not there is an underlying lung disease, a spontaneous pneumothorax can be primary or secondary [10]. On the other hand, pneumomediastinum may be either primary or secondary, depending on whether the cause is idiopathic or if an etiology, such as traumatic or iatrogenic has been identified [11]. Recently, there has been an increase in cases of pneumothorax, pneumomediastinum and subcutaneous emphysema, in confirmed COVID-19 patients, even more in those who have been intubated, raising the question of whether this is due to viral infection or a complication of the emergency procedure. According to several reports, only 1-2% of COVID-19 patients developed pneumothorax [6,12]. Although the underlying mechanism is unclear, the pathophysiological mechanism proposed is a diffuse alveolar damage, which leads to alveolar rupture and air leak [13]. However, our patient described here developed SPP, SPM, SCE, and acute bilateral pulmonary embolism prior to admission in the intensive care unit while on a high flow nasal cannula. In a case series of three COVID-19-patients who developed pneumomediastinum prior to intubation, two underwent autopsy, the possible mechanism suggested was direct viral damage with an increased inflammatory response [14].

The most common signs are chest pain and dyspnea [10]. Pneumothorax and pneumomediastinum are more frequent in males. However, while pneumothorax occurs mainly at rest [15], intense physical activity has been linked to pneumomediastinum occurrence [16]. The reported predisposing factors of these complications are drug misuse, asthma and other lung diseases with smocking [11,17]. Our patient was not an active smoker and did not have any other risk factor in developing these complications. Rupture of emphysematous bulla could be one of the causes of developing pneumothorax which could lead to subcutaneous emphysema. The therapeutic approach remains challenging; once SPP, SPM were diagnosed, the patient should be closely monitored to avoid respiratory deterioration. Pneumomediastinum, pneumothorax, and SCE are uncommon COVID-19 findings that may suggest a poor prognosis for patients and may also be associated with high morbidity as well as a more extended hospital stay. They can be considered as COVID-19 itself, as well as the complication of management of COVID-19. Our patient also presented bilateral acute pulmonary embolism within admission in addition to SPP, SPM and SCE. Several studies have reported the increased prevalence of thrombotic events in patients infected by the SARS-CoV-2 [17]. Although the pathogenesis remains unknown, the systemic inflammation, endothelial dysfunction, in addition to the hypercoagulable state, are mechanisms that most authors have proposed to explain the occurrence of these thromboembolic events in COVID-19 patients. Besides, hypoxemia in critical ill, further increased blood viscosity [18]. Therefore, it is essential to identify patients at a high risk of these thromboembolic events for an adequate thromboprophylaxis with heparins which have been shown to decrease mortality in these COVID-19 patients [19].

## Conclusion

The exact underlying pathogenesis of COVID-19 infection leading to thrombotic disorders, pneumothorax, pneumomediastinum, and SCE is not yet understood. However, this unique case emphasizes the possible extrapulmonary complications in COVID-19-patients which may be present at the patient's initial presentation. Therefore, the physician should maintain a high clinical suspicion with good history taking and examination skills. Computed tomography chest imaging may help assess coexisting pathologies and complications.
